# Is it time to revisit the scoring of slow wave (N3) sleep?

**DOI:** 10.1093/sleep/zsaf063

**Published:** 2025-03-13

**Authors:** Shaun Davidson, Rachel Sharman, Simon D Kyle, Lionel Tarassenko

**Affiliations:** Department of Engineering Science, Institute of Biomedical Engineering, University of Oxford, Oxford, UK; Nuffield Department of Clinical Neurosciences, Sir Jules Thorn Sleep and Circadian Neuroscience Institute, University of Oxford, Oxford, UK; Nuffield Department of Clinical Neurosciences, Sir Jules Thorn Sleep and Circadian Neuroscience Institute, University of Oxford, Oxford, UK; Department of Engineering Science, Institute of Biomedical Engineering, University of Oxford, Oxford, UK

**Keywords:** slow wave sleep, sleep and the brain, EEG analysis, gender, mathematical modeling

## Abstract

The use of a fixed electroencephalogram (EEG) amplitude threshold of 75 µV for labeling slow waves is a subject of ongoing discussion given EEG amplitude is known to vary with age and sex. This paper investigates the impact of this amplitude threshold on age- and sex-related trends in visually annotated slow wave sleep (SWS). Automated methods for labeling SWS using data-driven thresholds and amplitude- or frequency-based inputs are developed. Age- and sex-related trends in SWS derived from visual annotation and automated labeling are then compared across a cohort of 2913 participants from the Sleep Heart Health Study.

In the selected cohort, males exhibit an age-related decrease in visually annotated SWS, which is preserved when using automated labeling. In contrast, females exhibit a mild age-related increase in visually annotated and amplitude-labeled SWS, but an age-related decrease in frequency-labeled SWS. Furthermore, using frequency-labeled SWS results in a reduction in SWS in females to a level comparable to that of males. Overall, the consistency of age-related trends in SWS in males between visual annotation and automated labeling, as well as the lack of consistency in these trends in females, is striking. Given that the 75 µV amplitude threshold was established using data acquired primarily from young males, these results suggest that observed sex-based differences in visually annotated SWS may be artifactual rather than physiological, and a result of the 75 µV amplitude criterion. This sex-related disparity highlights the need for the American Association of Sleep Medicine guidelines for scoring SWS to be reviewed and updated to provide equivalent performance for males and females.

## Graphical Abstract

**Graphical Abstract:**
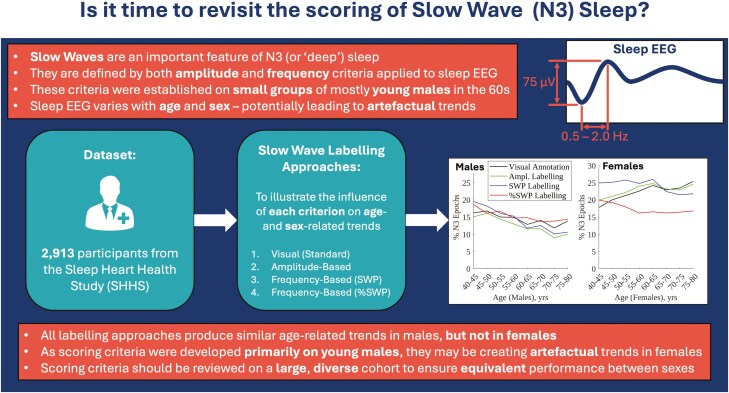

**Statement of Significance**

Slow wave sleep (SWS) is an important sleep stage, but the labeling of slow waves using a fixed electroencephalogram (EEG) amplitude threshold has long been a subject of debate as EEG amplitude is known to vary with age and sex. This study investigates the impact of this fixed amplitude threshold on age- and sex-related trends in SWS across a large, retrospective dataset. The analysis involves comparing trends in visually annotated slow wave sleep with trends derived using an automated method for labeling SWS and either amplitude- or frequency-based criteria. Results suggest that observed sex-based differences in visually annotated SWS may be artifactual rather than physiological, and a result of the amplitude threshold.

Despite there being well-established differences between male and female physiology, these differences are not always taken into account in biomedical research or medical diagnostic guidelines [1–3]. While the National Institute for Health (NIH) mandated in 1993 (after several “high profile male-only heart trials undertaken in the 1970s and 1980s” [4]) that trials be conducted such that it could be assessed whether “variables being studied... affect women... differently than other subjects” [5], many diagnostic metrics and thresholds were established well before this and have not necessarily undergone significant subsequent revisions. For example, the association between body mass index (BMI), a widely used index that has remained relatively unchanged since the 1850s, and the percentage of body fat is known to be significantly different between males and females [6]. Despite this, identical thresholds are used for classifying males and females as underweight, overweight, or obese. Similarly, there are well-established differences between blood pressure in males and females, and research has suggested that lower thresholds should be used to diagnose hypertension in females than in males [7]. Despite this, identical thresholds are used for diagnosing hypertension in both sexes.

Another area of research for which such sex differences are relevant is sleep. There is ongoing research into the association between sleep and health outcomes [8, 9]. Sleep can be divided into stages defined primarily, but not exclusively, by electroencephalogram (EEG) characteristics, including N1 (light), N2 (intermediate), N3 (slow wave), and rapid eye movement (REM) sleep. Sleep follows a typical topography, with periodic cycles of REM sleep that increase in duration throughout the night, and more N3 sleep earlier in the night [10]. A sleep stage of particular interest is N3 or slow wave sleep (SWS), which is an important sleep stage associated with memory reinforcement, immune function, and repair of tissues [11]. Reduction in the duration of this sleep stage has been shown to be associated both epidemiologically with type 2 diabetes and acutely with reduced glucose tolerance [12, 13]. Further, recent studies have shown that reduced SWS duration is associated with an increased risk of dementia and that SWS duration may thus “be a modifiable dementia risk factor” [14].

Current American Association of Sleep Medicine (AASM) guidelines define “slow waves” in the EEG as waves with a frequency of 0.5–2.0 Hz and a minimum amplitude of 75 µV, with N3 sleep defined by the presence of at least 20% slow waves in a 30-second ‘epoch’ of sleep [15]. However, there are known issues with the application of these guidelines. EEG amplitude reduces with age [16–18], with the result that the 75 µV threshold can cause epochs in older participants not to be “scored as SWS but... rather assigned to stage 2 sleep—although all other characteristics of the sleep epoch suggest the presence of SWS (i.e. the predominance of low-frequency EEG waves but the absence of eye movements along with reduced muscle tone)” [19]. Further, EEG amplitude and signal quality are known to vary with hair thickness, volume, and type [20]; thus amplitude thresholds developed on a specific ethnicity or sex may not be broadly applicable to other groups. Finally, and more generally, there are sufficient differences between the EEG in males and females that it is possible to train models to determine sex from the EEG to a high degree of accuracy [21, 22].

These differences in EEG characteristics between younger and older males and females were investigated through a series of data-driven experiments in a recent publication [23]. The authors employed a training dataset and an independent test set of EEG recordings, with slow wave density (number of slow waves per minute of NREM sleep) as the main metric for their analyses. They concluded that the well-known decrease in SWS with age, widely reported in the literature [19, 24, 25], was valid, but that sex-related differences in SWS were related to EEG amplitude, with older males more likely to produce low-amplitude slow waves in comparison to older females and younger adults.

The predecessor of the current AASM guidelines for sleep annotation was a manual by Rechtschaffen and Kales (R & K) [26], which was “essentially a formalization” [18] of the sleep stages previously described by Dement and Klietman in [27] (a study which, notably, consisted of 26 males and 7 females, most of whom were between the ages of 20 and 30 years). The only difference between current AASM guidelines for identifying slow waves and those in the R & K manual is the change in the definition of slow wave frequency from 0.5–4.0 Hz (in R & K) to 0.5–2.0 Hz that was introduced in the 2014 AASM guidelines (v2.1) [28]. Importantly, the R & K manual states that “alternative measures of slow wave activity might have a usefulness and empirical significance not enjoyed by the measure chosen” and that “this should not deter investigators from using measures of slow wave activity other than the one suggested here.” As noted by [18], “The guidelines of Rechtschaffen and Kales were meant as a reference method. However, it became, unintentionally, a gold standard. The rules have never been appropriately validated.”

In line with this, a recent publication [29] notes, “The R & K manual and the current AASM manual do not provide an explanation for the 20% of epoch rule or the 75 µV amplitude rule.” The authors further note that the absence of an empirical rationale for these thresholds makes providing justification for any particular alternative threshold challenging, and acknowledge that there is value in maintaining continuity with the thousands of existing sleep-related publications that employ these thresholds. Regardless [29], stresses that “measures of sleep continuity and depth that are reliable and clinically relevant should be a focus of clinical research.”

Fundamentally, the R & K thresholds were “designed for paper recordings including specifications for filters, gains, paper speed, pen deflection, number of channels..” [18]., in which context the 75 µV threshold makes sense as a surrogate for low-frequency wave power that can be easily and rapidly visually applied. However, with the move to digitized EEG, it is possible to directly compute wave power in any given frequency band using frequency-based methods. Using these frequency-based methods, a continuous association has been shown between sleep depth (as measured by audio volume required to trigger arousal) and absolute delta power (0.5–4.0 Hz), with no notable step change in sleep depth when wave amplitude exceeds a 75 µV threshold [30]. Indeed, it was noted as far back as the 1970s that “if future standards for measurement of slow wave activity were based upon spectral intensity criteria rather than the present visual criteria, perhaps a more reliable and sensitive indicator of changes in SWS with age, drugs or illness might be possible” [31].

This paper investigates the impact of the 75 µV amplitude threshold on age- and sex-related trends in visually annotated SWS (i.e. N3 sleep) by comparing these trends with those derived using both amplitude- and frequency-based methods for automated labeling of SWS. The automated labeling approach employed in this paper allows for continuous, second-by-second labeling of SWS using data-driven thresholds and EEG envelope amplitude, slow wave power (SWP), or percentage slow wave power (%SWP) as input. By comparing age- and sex-related trends in SWS derived using visual annotation with those derived using automated labeling, insight can be provided into the influence of current AASM thresholds on the age- and sex-related trends in SWS reported in the literature.

## Methods

### Dataset: the Sleep Heart Health Study

The Sleep Heart Health Study (SHHS) was designed to investigate the association between sleep-disordered breathing and cardiovascular health [32]. This multi-center study in the United States recruited 6441 participants from existing cardiovascular disease studies to its first phase (SHHS1) between November 1995 and January 1998. Cardiovascular outcomes, including coronary heart disease, stroke, all-cause mortality, and hypertension, were monitored until 2011. Inclusion criteria for participants were being at least 40 years of age, having no history of sleep apnea treatment, not having undergone a tracheotomy, and not currently receiving home oxygen therapy. Given the interest in sleep-disordered breathing, snorers were oversampled during recruitment.

The SHHS database contains 2-channel EEG (C3/A2 and C4/A1), sampled at 125 Hz. Hardware filtering consisted of a high-pass filter with a cut-off at 0.15 Hz and no low-pass filter. Documentation for the study [32] notes that the C4/A1 channel “visually appeared to be a `cleaner’ signal (less high frequency within the waveforms) than C3/A2.” Furthermore, while AASM guidelines state that ideally frontal electrodes (F4/A1 or F3/A2) should be used to assess slow waves, the guidelines note that “when using the acceptable EEG derivations... EEG amplitude to determine slow wave activity should be measured using the C4-M1 (i.e. C4/A1) derivation” [15]. For these reasons, the C4/A1 EEG channel was selected for the analysis conducted in this paper, all of which were performed using MATLAB R2023b.

EEG records in the SHHS1 database were excluded from analysis if:

1. The participant met the AASM threshold for moderate obstructive sleep apnea (i.e. apnea–hypopnea index (AHI) > 15 [33]). This criterion was applied to mitigate the potential effects of the over-representation of sleep-disordered breathing (due to oversampling of snorers during recruitment) in the database. Note that short- and mid-term follow-up questionnaires (Supplementary Appendix B) found low rates of other diagnosed sleep disorders (e.g. insomnia, restless leg syndrome, and narcolepsy) in the selected cohort, and exclusions were therefore not made based on these conditions.
2. The participant was greater than 80 years of age, due to the small number of participants over 80 in the database.
3. Greater than 2% of NREM EEG epochs were classified as outliers using log-transformed standard deviation, as implemented as a signal quality metric in [34]. This criterion was applied to exclude participants with a noisy NREM C4/A1 EEG signal.
4. The C4/A1 EEG was otherwise difficult to visually annotate, resulting in the C3/A2 EEG being used for manual sleep annotation (Supplementary Appendix A).

### Automated labeling of SWS

AASM guidelines define slow waves as having a frequency of 0.5–2.0 Hz and a minimum amplitude of 75 µV. During visual annotation, a 30-second “epoch” of sleep is labeled as N3 sleep if it contains at least 20% (6 seconds) of these slow waves [15]. As such, the AASM guidelines include frequency (0.5–2.0 Hz), amplitude (75 µV), and temporal (6 seconds of slow waves in a 30-second epoch) criteria. During visual annotation, these criteria interact to produce the sex- and age-related trends in N3 sleep reported in the literature. To investigate the influence of each criterion on these trends, automated labeling methods that emphasized or de-emphasized the influence of these criteria were developed. These methods were then applied and any differences in the resulting age- and sex-related trends in SWS observed.

AASM sleep staging involves first identifying slow waves in a continuous fashion using amplitude (75 µV) and frequency (0.5–2.0 Hz) criteria. To emulate this approach, while separating amplitude and frequency criteria, two automated, continuous methods for labeling slow waves were developed. The first of these methods is a time-domain method that relies upon an amplitude criterion, and the second is a frequency-domain method that relies upon a power or percentage power criterion.

#### Amplitude-based approach.

Amplitude-based labeling of SWS was performed using an envelope method as follows:

1. Bandpass filter the C4/A1 EEG within the broad range of physiologically relevant frequencies (0.5 Hz–35.0 Hz, encompassing the delta to gamma bands, as in [34]).
2. Perform half-wave rectification on the EEG signal and an inverted copy of the EEG signal.
3. Low-pass filter the (non-inverted) rectified EEG signal using an eighth-order Butterworth filter with a cutoff frequency of 2.0 Hz to create the upper envelope (which follows the peaks of the EEG signal).
4. Low-pass filters the inverted, rectified EEG signal using an eighth-order Butterworth filter with a cutoff frequency of 2.0 Hz. Invert the resulting signal to create the lower envelope (which follows the troughs of the EEG signal).
5. Compute the peak-to-trough envelope amplitude by taking the difference between the upper and lower envelope signals.

**Figure 1.**
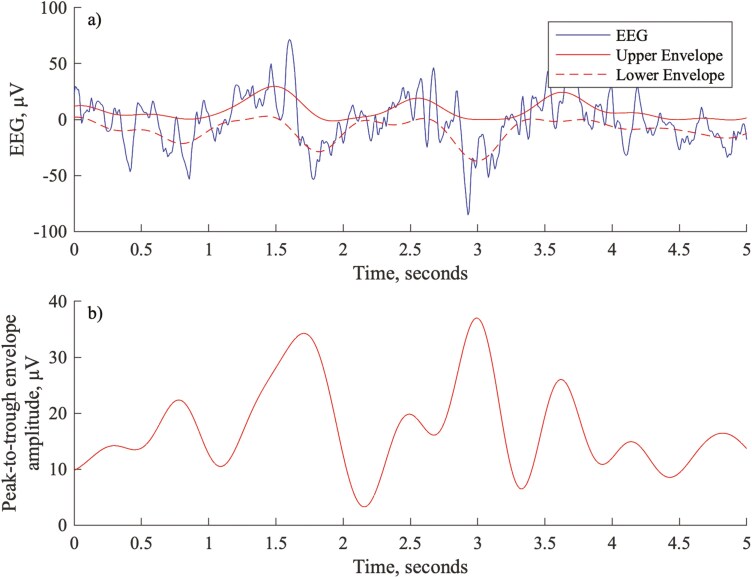
Example EEG and corresponding envelope function for a segment of visually annotated N3 sleep: (A) EEG with corresponding upper and lower envelope; (B) Amplitude of the corresponding envelope function.

An example of the envelope signal produced using this approach on a segment of visually annotated N3 sleep EEG is shown in Figure 1. The 2.0 Hz low-pass filter corresponds to the maximum frequency of a slow wave according to AASM criteria and thus smooths the resulting envelope such that slow waves (for which peaks and associated troughs are separated by at least 0.5 seconds) produce a sustained period of high envelope amplitude. However, the low-pass filter also significantly reduces the signal amplitude relative to the underlying EEG, hence the AASM 75 µV criterion is no longer applicable for labeling SWS using this envelope signal.

#### Frequency-based approach.

Frequency decomposition of the EEG was performed using Welch’s method, the “gold standard” method for this task [12, 34]. Welch’s method was developed to reduce noise in periodograms of non-stationary signals by averaging multiple periodograms generated over short segments of overlapping Hamming-windowed data. A modified Welch’s method approach using rolling overlapping windows was developed to provide a continuous, second-by-second assessment of EEG spectral power. This approach was implemented as follows:

1. Bandpass filter the C4/A1 EEG within the broad range of physiologically relevant frequencies (0.5 Hz–35.0 Hz) [12].
2. Compute periodograms for 4-second Hamming-windowed segments of EEG, sliding by 1 second (i.e. with a 75% overlap between windows). While it is more typical to employ a 50% overlap between windows, a 75% overlap produces a new periodogram for every 1 second of signal (as opposed to every 2 seconds with a 50% overlap). This is beneficial when averaging periodograms over short signal segments as it means that a given signal segment produces a greater number of periodograms to be averaged, hence reducing noise in the output periodogram.
3. Implement Welch’s method by calculating a rolling 5-window average of the periodograms, giving a second-by-second output periodogram which, at a given point in time, is dependent on the neighboring 4 seconds of EEG on each side of that point (primarily the neighboring second on either side, as shown in Figure 2).

**Figure 2.**
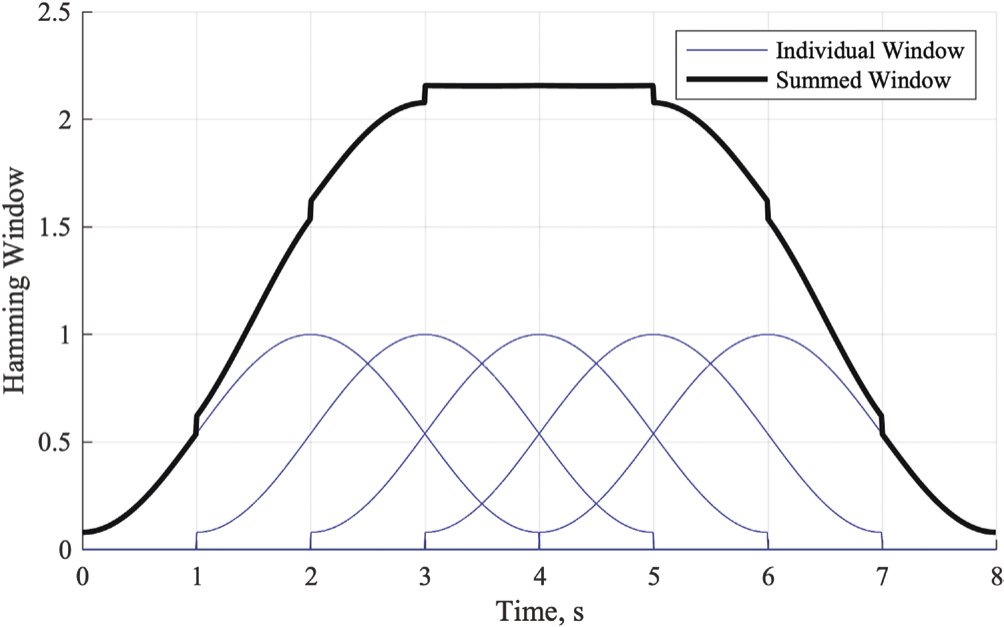
Summed Hamming window values for 4-second overlapping windows sliding by 1 second.
4. Use either:
**Slow Wave Power (SWP) Method:** Calculate the band power in the slow wave (0.5–2.0 Hz) band.
**Percentage Slow Wave Power (%SWP) Method:** Calculate relative band power in the slow wave (0.5–2.0 Hz) band by computing the ratio of SWP to the total power observed in the 0.5–35 Hz frequency range.

#### Establishing thresholds for labeling SWS.

The amplitude-based, SWP frequency-based, and %SWP frequency-based methods developed thus far each provide a continuous index associated with SWS, but for each method, an appropriate threshold must be selected for labeling SWS. Using AASM guidelines, after slow waves are identified (using frequency and amplitude criteria), a 30-second epoch of sleep is visually annotated as N3 sleep if it contains at least 6 seconds of slow waves, and these visual annotations of N3 sleep are available in the SHHS1 database. As such, initial thresholds for the automated labeling of SWS were determined by assessing the duration of amplitude- or frequency-labeled SWS present in visually annotated epochs in the SHHS database across a range of different thresholds.

For this exercise, visually annotated N2 and N3 epochs from the selected SHHS cohort were employed. Note that visual annotation in the SHHS database was performed according to R & K guidelines, including a distinct N4 sleep stage. For the purpose of the analyses in this paper, N4 sleep was merged with N3 sleep in accordance with AASM guidelines. For a given amplitude, SWP, or %SWP threshold, each N2 or N3 epoch was then considered as having been identified “correctly” (i.e., corresponding to AASM guidelines) if:

A (visually annotated) N2 sleep epoch contained less than 20% (6 seconds) of amplitude- or frequency-labeled SWS. Note that while the definition of N2 sleep also refers to other sleep features (K-complexes and spindles), these features are used to identify N2 sleep “in the absence of criteria for N3 (sleep),” with it being noted that “sleep spindles may persist in stage N3 sleep” [15].
A (visually annotated) N3 sleep epoch contained greater than 20% (6 seconds) of amplitude- or frequency-labeled SWS.

For each amplitude, SWP, or %SWP threshold, the overall percentage of epochs identified correctly was then calculated, and the threshold corresponding to the greatest percentage of correctly-identified N2 and N3 epochs was selected for automated labeling of SWS.

While such an approach does mean that the thresholds selected for frequency-based labeling of SWS are dependent on existing visual annotation criteria, which include an amplitude criterion, this approach was selected for the simple reason that the only reference labels available in the SHHS dataset are visual annotations. To characterize the dependence of resulting trends in SWS on the choice of threshold used for automated labeling, a sensitivity analysis was conducted. This analysis involved repeating the automated labeling process using reduced (90% of optimal) and increased (110% of optimal) thresholds for labeling.

### Assessing age- and sex-related trends

Having established thresholds for the continuous, automated amplitude- and frequency-based labeling of SWS, the following population trends were evaluated, with participants grouped by sex and stratified by age in 5-year bins:

- Demographics and visual annotation.

1. Number of participants.
2. Total sleep time, as determined by visual annotation.
3. % overnight duration of (visually annotated) N3 sleep.

- Amplitude-based labeling.

1. Envelope amplitude during (visually annotated) NREM sleep.
2. % overnight duration of continuous, amplitude-labeled SWS.

- SWP frequency-based labeling.

1. SWP during (visually annotated) NREM sleep.
2. % overnight duration of continuous, SWP frequency-labeled SWS.

- %SWP frequency-based labeling.

1. %SWP during (visually annotated) NREM sleep.
2. % overnight duration of continuous, %SWP frequency-labeled SWS.

Linear trend coefficients, R^2^ values, and F-test statistics were computed for age-related trends in the population means of each metric, with *p* < .05 used as the threshold for establishing a significant linear trend. Whether there were significant differences between linear trends in males and females for a given metric was assessed as in [35], using a two-tailed Student’s T-test and a threshold for significance of *p* < .05.

### Comparing visual, frequency-based, and amplitude-based annotation of NREM epochs

According to AASM guidelines, a 30-second “epoch” of sleep is annotated as N3 sleep if it contains at least 6 seconds of slow waves. Using this criterion to re-annotate N2 and N3 epochs using continuous amplitude- or frequency-labeled SWS allows for a more direct comparison to be made between visual annotation and amplitude- and frequency-based annotation, as visual annotation in SHHS1 exists only at the epoch level. Re-annotation of (visually annotated) N2 and N3 epochs was performed as follows:

- **Amplitude-based:** A given epoch was annotated as N3 if it contained at least 6 seconds of amplitude-labeled SWS (i.e. 6 seconds of envelope amplitude above the threshold established for labeling SWS), and N2 otherwise.
- **Frequency-based:** A given epoch was annotated as N3 if it contained at least 6 seconds of frequency-labeled SWS (i.e. 6 seconds of SWP or %SWP above the threshold established for labeling SWS), and N2 otherwise.

Once again, population trends were evaluated, with participants grouped by sex and stratified by age in 5-year bins. As previously, linear trend coefficients, R^2^ values, and F-test statistics were computed for age-related trends in the population means of each metric, and linear trends between males and females were compared [35].

### Spectral analyses

As an extension of the frequency-based analysis, and to provide insight into age- and sex-related trends in other EEG spectral bands, averaged periodograms for males and females in the youngest (40–50) and oldest (70–80) decile in the selected SHHS cohort were compared. Periodograms were computed both for raw power and percentage power in the following bands (similar to [12]):

- Slow: 0.5–2.0 Hz.
- Delta (excluding Slow): 2.0–4.0 Hz.
- Theta: 4.0–8.0 Hz.
- Alpha: 8.0–12.0 Hz.
- Sigma: 12.0–16.0 Hz.
- Beta: 16.0–30.0 Hz.
- Gamma: 30.0–35.0 Hz.

Periodograms were computed both for the entire night’s (visually annotated) NREM sleep and for (visually annotated) N3 sleep. Differences in band power between deciles were assessed using a two-tailed *t*-test, with a threshold for significance of *p* < .05.

## Results

### Demographics and visually annotated metrics

Of the 6441 participants in the SHHS1 dataset, 3127 did not meet the criteria for moderate obstructive sleep apnea. Of these 3127, 95 were excluded due to poor EEG signal quality and 119 due to being over the age of 80, leaving 2913 records for analysis. Table 1 provides a summary of demographics, including co-morbidities and use of medication, for this cohort. Participants are generally older and slightly overweight, with 29.9% of the cohort having hypertension and 33.5% taking anti-hypertensive medication. Females are overrepresented, due to a greater prevalence of obstructive sleep apnea in males within the SHHS1 dataset, as in the general population [36]. Further demographic information, including age-related trends in AHI and arousal index (AI), as well as information on the prevalence of other sleep disorders in the selected SHHS1 cohort, is provided in Supplementary Appendix B.

**Table 1.** Overview of Cohort Demographics, Co-morbidities, and Common Medication

Demographics	Male	Female	Overall
Participants, # (%)	1031 (35.4)	1882 (64.6)	2913 (100)
Age (years), mean (SD)	60.2 (10.6)	60.2 (10.4)	60.2 (10.5)
BMI, mean (SD)	27.1 (3.7)	27.1 (5.1)	27.1 (4.6)
AHI, mean (SD)	8.6 (3.8)	7.0 (3.9)	7.5 (3.9)
AI, mean (SD)	15.5 (6.9)	14.5 (6.5)	14.8 (6.6)
Hypertension, # (%)	316 (30.6)	556 (29.5)	872 (29.9)
Cardiovascular Disease+, # (%)	114 (11.1)	111 (5.9)	225 (7.7)
Diabetes, # (%)	68 (6.6)	69 (3.7)	137 (4.7)
Anti-hypertensive, # (%)	340 (33.0)	637 (33.8)	977 (33.5)
ACE Inhibitors, # (%)	152 (14.7)	179 (9.5)	331 (11.4)
Anti-depressants, # (%)	44 (4.3)	185 (9.8)	229 (7.9)
Benzodiazepines, # (%)	46 (4.5)	130 (6.9)	176 (6.0)

+Defined as prior history of stroke, myocardial infarction, congestive heart failure, or angina.

**Figure 3.**
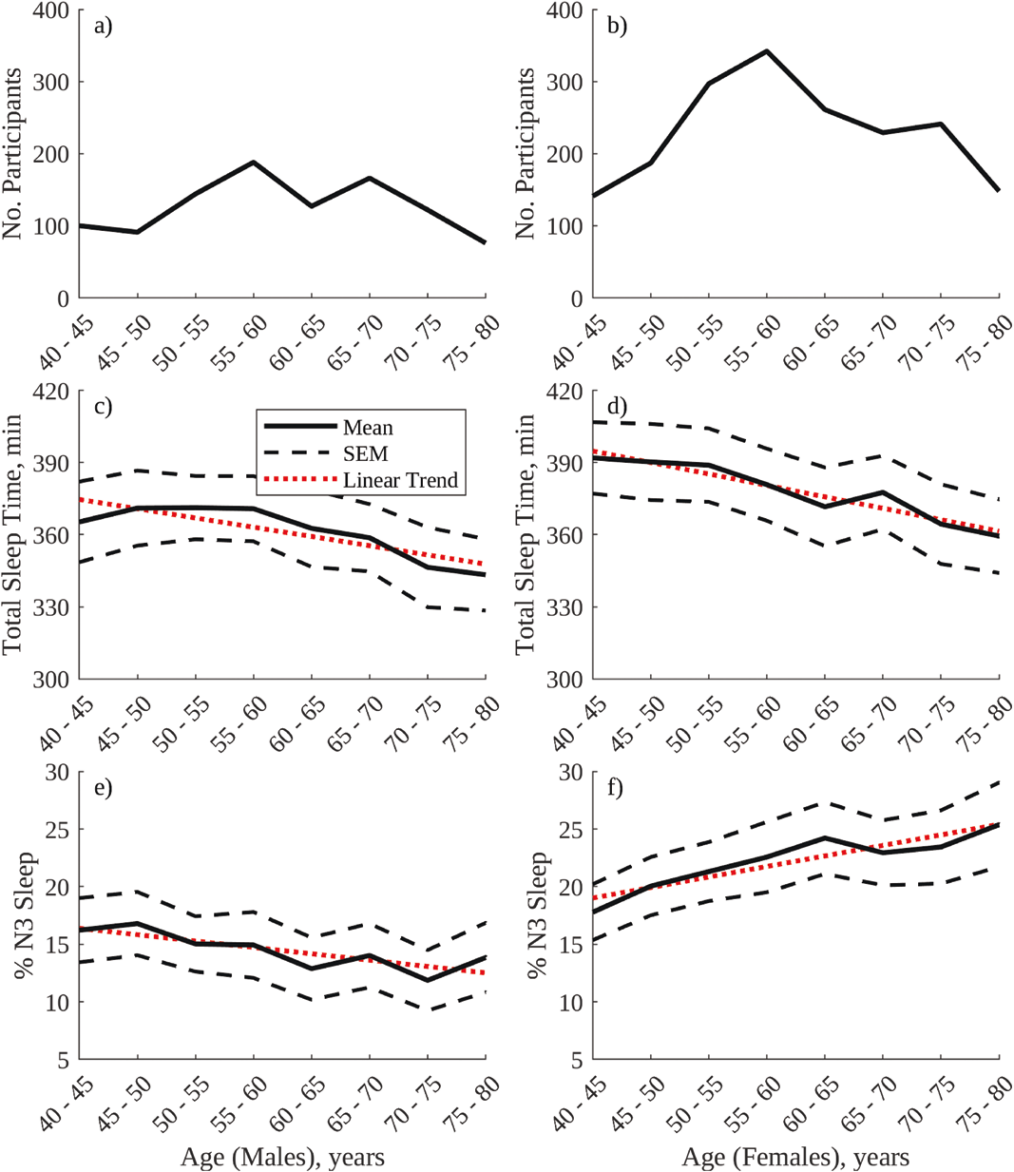
Summary of visually annotated sleep metrics grouped by age for males and females in the SHHS1 database: (A) Number of participants, males; (B) Number of participants, females; (C) Total sleep time, males; (D) Total sleep time, females; (E) % overnight duration of visually annotated N3 sleep, males; f) % overnight duration of visually annotated N3 sleep, females.

Figure 3 shows the number of participants, visually annotated total sleep time, and % overnight duration of visually annotated N3 sleep for each sex and age group in the selected cohort of SHHS1 participants. Table 2 provides accompanying linear trend statistics. In Figure 3, there are more females than males in each age group due to the exclusion of participants with moderate sleep apnea (as in Table 1), and the most populated age band for both sexes is the 55–60 band. Total sleep time (panels c and d) is greater in females than in males, on average, and decreases with age for both sexes. % visually annotated N3 sleep (panels e and f) shows a significant decrease with age in males, which is well documented in the literature [25, 37, 38], but an increase with age in females, for whom less consistent age-related trends in % visually annotated N3 sleep are reported in the literature. It is worth noting here that the age range of participants in the SHHS database (40–80) does not include the “young” 20–30-year-old demographic employed in some studies [25] and that either no change or an increase with age in % visually annotated N3 sleep in females has been reported in prior publications using the same [37] and other [38] datasets across a similar age range. Finally, it is worth recalling that the trends in Figure 3 are derived from the gold-standard visual annotation of PSG in the sleep lab, as opposed to any novel approach.

**Table 2.** Linear Trend Statistics for Visually Annotated Sleep Metrics in Figure 3

Metric	R^2^	*P*	Constant	Gradient
Total sleep time, males	0.73	.01	378.48^*^	−3.84
Total sleep time, females	0.91	2.06E-4	399.55^*^	−4.76
%N3 sleep, males	0.68	.01	16.93	−0.55^*^
%N3 sleep, females	0.85	1.20E-3	18.10	0.91^*^

^*^Denotes a significant difference (*p* < .05, 2-tailed *t*-test) in linear trend parameters between males and females.

### Continuous amplitude-based labeling

**Figure 4.**
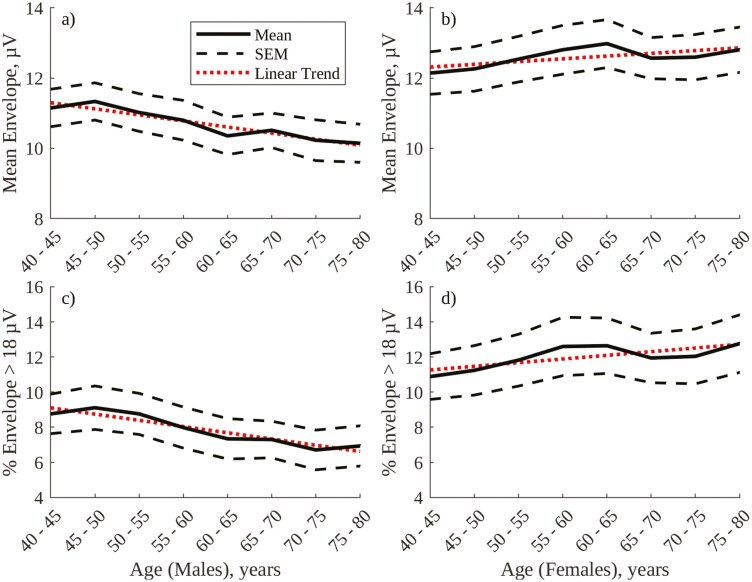
Amplitude annotation sleep metrics, grouped by age for males and females in the SHHS1 database: (A) Mean overnight NREM envelope amplitude, males; (B) Mean overnight NREM envelope amplitude, females; (C) % overnight duration of envelope amplitude >18 µV, males; (D) % overnight duration of envelope amplitude >18 µV, females.

The optimal threshold for continuous, amplitude-based labeling of SWS was found to be 18 µV, at which threshold there is an 87.0% agreement between visual and amplitude-based annotation of N2 and N3 epochs (see figure C1 in Supplementary Appendix C and sensitivity analysis in Supplementary Appendix D). Figure 4 shows age- and sex-related trends in mean NREM envelope amplitude and % overnight duration of SWS labeled using the 18 µV threshold, with Table 3 providing associated linear trend statistics. In line with visually annotated % N3 sleep in Figure 3, both mean NREM envelope amplitude (panels a and b) and % amplitude-labeled SWS (panels c and d) decrease significantly with age in males but increase with age in females (with the increase in % amplitude-labeled SWS in females being statistically significant).

**Table 3.** Linear Trend Statistics for Amplitude- and Frequency-Based Sleep Metrics in Figures 4–6

Metric	R^2^	*p*	Constant	Gradient
Envelope amplitude (µV), males	0.90	3.60E-4	11.47^*^	−0.17^*^
Envelope amplitude (µV), females	0.46	.06	12.23^*^	0.08^*^
%Envelope amplitude > 18 µV, males	0.89	4.73E-4	9.46^*^	−0.36^*^
%Envelope amplitude > 18 µV, females	0.57	.03	11.05^*^	0.21^*^
SWP (µV^2^), males	0.90	3.30E-4	132.59^*^	−5.22^*^
SWP (µV^2^), females	0.55	.04	167.05^*^	−2.43^*^
%SWP > 285 µV^2^, males	0.92	1.42E-4	9.97^*^	−0.69^*^
%SWP > 285 µV^2^, females	0.66	.01	13.74^*^	−0.26^*^
%SWP, males	0.00	.94	42.91	−0.01
%SWP, females	0.26	.20	43.52	0.09
%SWP > 70%, males	0.65	.02	8.79	−0.29
%SWP > 70%, females	0.61	.02	9.62	−0.23

^*^Denotes a significant difference (*p* < .05, 2-tailed *t*-test) in linear trend parameters between males and females.

### Continuous frequency-based labeling

**Figure 5.**
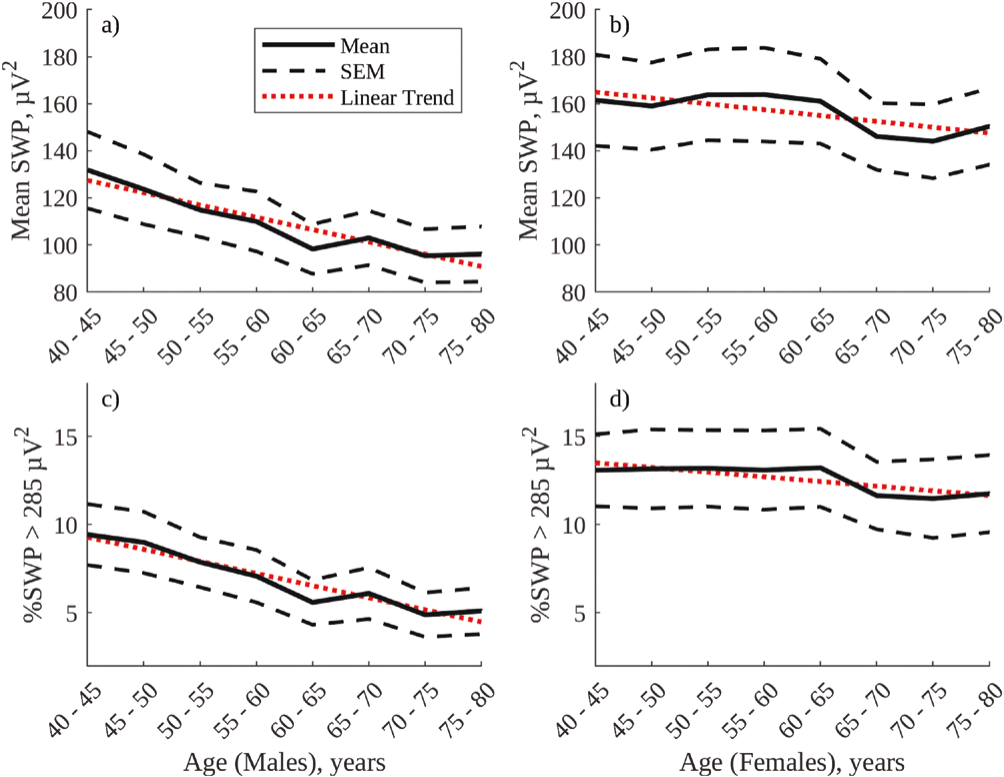
SWP frequency annotation metrics, grouped by age for males and females in the SHHS1 database: (A) Mean overnight NREM SWP, males; (B) Mean overnight NREM SWP, females; (C) % overnight duration of SWP > 285 µV^2^, males; (D) % overnight duration of SWP > 285 µV^2^, females.

The optimal threshold for continuous, SWP frequency-based labeling of SWS was found to be 285 µV^2^, at which threshold there is an 84.8% agreement between visual and frequency-based annotation of N2 and N3 epochs (see figure C2 in Supplementary Appendix C and sensitivity analysis in Supplementary Appendix D). Figure 5 shows age- and sex-related trends in mean NREM SWP and % overnight duration of SWS labeled using the 285 µV^2^ threshold. Table 3 provides accompanying linear trend statistics. In contrast to visually annotated % N3 sleep in Figure 3 and amplitude annotated SWS in Figure 4, both mean NREM SWP (panels a and b) and %SWS as labeled using SWP (panels c and d) show significant decreases with age in both males and females. However, in line with Figures 3 and 4, the % overnight SWS is notably greater in females than in males.

**Figure 6.**
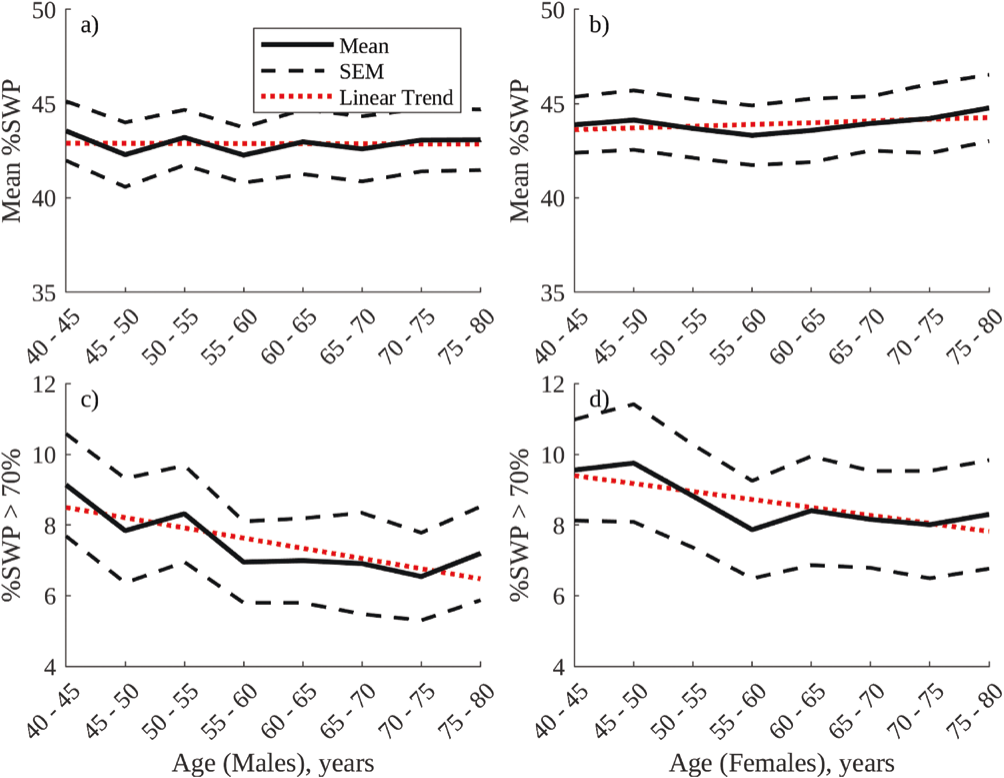
%SWP frequency annotation metrics, grouped by age for males and females in the SHHS1 database: (A) Mean overnight NREM %SWP, males; (B) Mean overnight NREM %SWP, females; (C) % overnight duration of %SWP > 70, males; (D) % overnight duration of %SWP > 70, females.

The optimal threshold for continuous, %SWP frequency-based labeling of SWS was found to be 70%, at which threshold there is an 81.4% agreement between visual and frequency-based annotation of N2 and N3 epochs (see figure C3 in Supplementary Appendix C, and sensitivity analysis in Supplementary Appendix D). Figure 6 shows age and sex-related trends in mean NREM %SWP and %SWS as labeled using the 70% SWP threshold. Table 3 provides accompanying linear trend statistics. Mean NREM %SWP (panels a and b) does not vary significantly with age in males or females, while %SWS as labeled using %SWP (panels c and d) decreases significantly with age in both males and females. Mean NREM %SWP and %SWS as labeled using %SWP do not exhibit a significant difference between males and females, in contrast to %SWS as labeled using envelope amplitude in Figure 4 or SWP in Figure 5.

### Epoch-by-epoch labeling of N3 sleep

**Figure 7.**
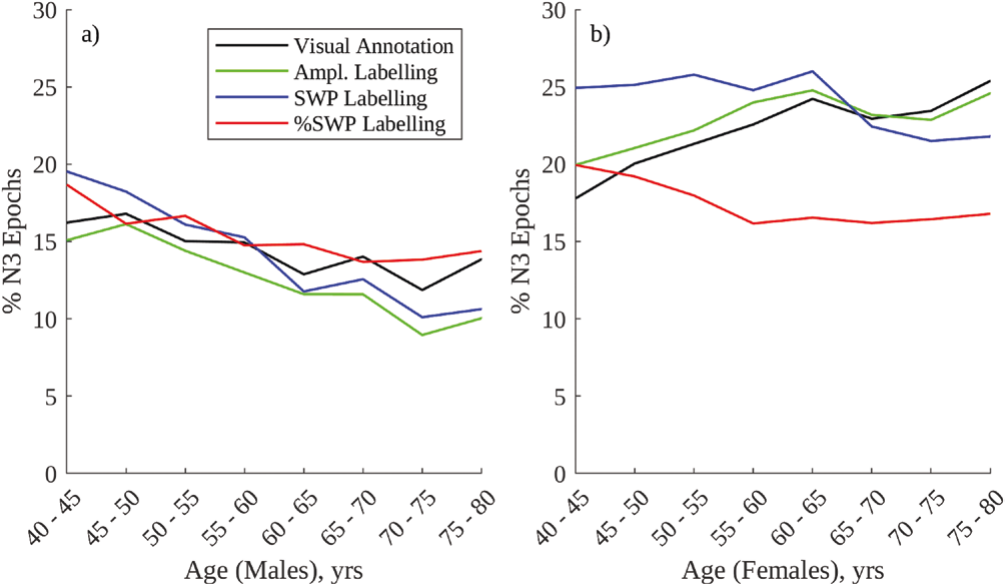
Comparison between visual, amplitude-, SWP frequency-, and %SWP frequency-based annotation of N3 sleep: (A) % overnight duration of N3 epochs, males; (B) % overnight duration of N3 epochs, females.

Figure 7 provides a comparison between visual, amplitude-based, SWP frequency-based, and %SWP frequency-based annotation of N2 and N3 epochs, and Table 4 provides accompanying linear trend statistics. Of note is the similarity between the age-related trends in %N3 sleep for males using all four methods, all of which show a significant decrease. This contrasts with trends observed in females, where frequency-based annotation shows a significant decrease in %N3 sleep with age, as in males, while amplitude-based and visual annotation show significant increases. Visual, amplitude and SWP frequency-based annotation approaches show greater %N3 sleep in females than in males, while %SWP frequency-based annotation does not exhibit a significant difference in the amount of N3 sleep between males and females.

**Table 4.** Linear Trend Statistics for N3 Sleep Metrics in Figure 7

Metric	R^2^	*p*	Constant	Gradient
%N3 sleep (visual), males	0.68	.01	16.93	−0.55^*^
%N3 sleep (visual), females	0.85	1.20E-3	18.10	0.91^*^
%N3 sleep (amplitude), males	0.89	4.36E-4	16.93^*^	−0.96^*^
%N3 sleep (amplitude), females	0.60	.02	20.40^*^	0.54^*^
%N3 sleep (freq-SWP), males	0.93	9.92E-5	20.54^*^	−1.39^*^
%N3 sleep (freq-SWP), females	0.61	.02	26.67^*^	−0.58^*^
%N3 sleep (freq-%SWP), males	0.76	4.96E-3	18.07	−0.60
%N3 sleep (freq-%SWP), females	0.66	.01	19.59	−.49

^*^Denotes a significant difference (*p* < .05, 2-tailed *t*-test) in linear trend parameters between males and females.

### Spectral analyses

**Figure 8.**
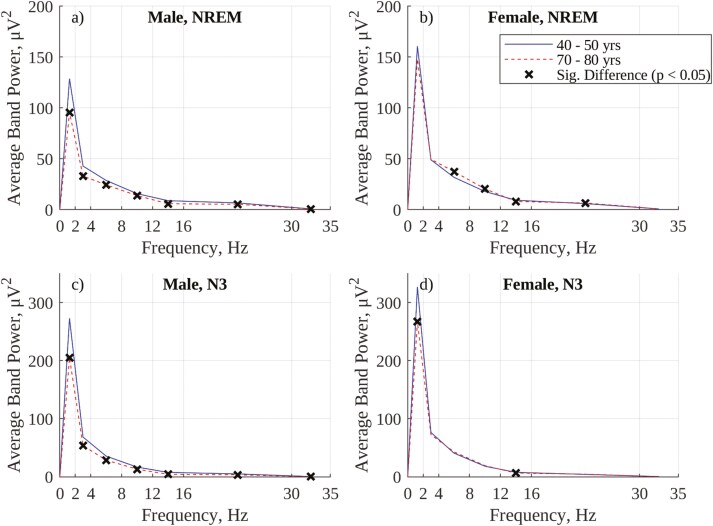
Comparison of absolute power periodograms across the full EEG frequency range for the youngest and oldest deciles in the selected dataset of SHHS1 patients: (A) Visually annotated NREM sleep, males; (B) Visually annotated NREM sleep, females; (C) Visually annotated N3 sleep, males; (D) Visually annotated N3 sleep, females.

To provide insight into age- and sex-related changes in EEG spectral behavior across the full range of the EEG spectrum (0.5 Hz to 35 Hz), periodograms of absolute power for the youngest (40–50) and oldest (70–80) deciles of participants, grouped by sex, were computed, as shown in Figure 8. Inspection of the absolute power periodograms for visually annotated NREM sleep (panels a and b) in the SWP range (0.5 Hz–2.0 Hz) show that females exhibit greater SWP than males and that there is no significant decrease in SWP between the ages of 40 and 80 in females, while there is in males. Further, males show a general decrease in power across most frequency bands between the ages of 40 and 80, while older females show a significant increase in some higher frequency bands (e.g. theta and alpha). Panels c and d show a similar decrease in power across most bands with age during visually annotated N3 sleep in males. Females, interestingly, show a significant decrease in SWP with age during visually annotated N3 sleep, despite such a decrease not being present during overall NREM sleep.

**Figure 9.**
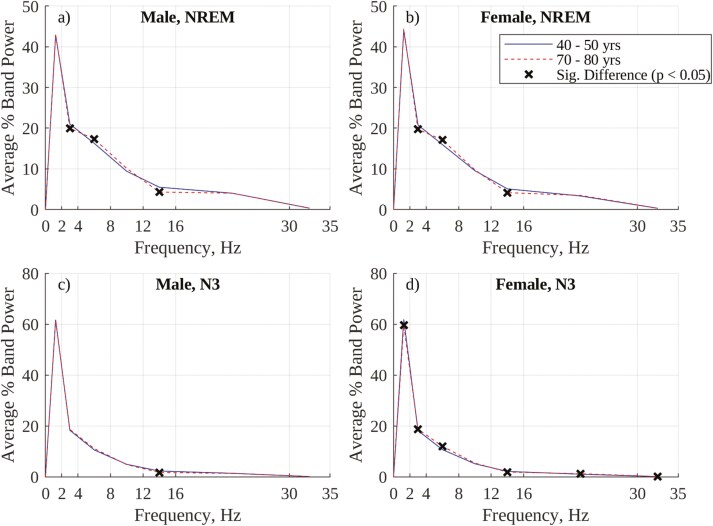
Comparison of percentage power periodograms for the youngest and oldest deciles in the selected dataset of SHHS1 patients: (A) Visually annotated NREM sleep, males; (B) Visually annotated NREM sleep, females; (C) Visually annotated N3 sleep, males; (D) Visually annotated N3 sleep, females.

Figure 9 shows periodograms for the same sex and age groups as in Figure 8 but computed using percentage (instead of absolute) power. In contrast to the broad decrease in power with age observed in males but not females in Figure 8 (using absolute SWP), the overnight NREM periodograms (panels a and b) show no significant change in %SWP with age for either sex, and identical changes for both sexes in higher frequency bands (a decrease in % delta power, an increase in % theta power, and a decrease in % sigma power). Interestingly, panels c and d show a significant decrease (*p* < .05, 2-tailed *t*-test) in %SWP during visually annotated N3 sleep in females but not in males.

## Discussion

There are two established differences in age-related trends in visually annotated N3 sleep between males and females. The first of these is that the overnight percentage of visually annotated N3 sleep decreases significantly with age in males but either increases (as in Figure 3 and [37]) or does not change (as in [39]) with age in females. However, using absolute SWP for frequency-based labeling of SWS (Figure 5) results instead in a significant decrease in the overnight percentage of SWS with age in females, as with males. This behavior may be a result of an SWP threshold consistently isolating power in the slow wave (0.5–2.0 Hz) band when labeling SWS, in contrast to an amplitude threshold which may be met by varying combinations of slow and higher frequency waves. The periodograms in Figure 8 show that older females have increased NREM theta and alpha power compared to younger females (a trend not present in males), and this increase in higher frequency EEG content may result in less consistent labeling of SWS using an amplitude threshold in these older females.

This possibility is reinforced by the fact that the periodograms in Figure 9 show no decrease in mean %SWP during NREM sleep with age in males or females, but show a significant decrease in mean %SWP during visually annotated N3 sleep with age in females that is not present in males. This result suggests that visual annotation of N3 sleep in older females is less consistent than in younger females, and includes epochs with reduced SWP, a trend that is not present in males. Further reinforcement is provided by the strong agreement between trends in visually annotated and amplitude-annotated N3 sleep (AASM slow wave frequency guidelines are incorporated here by applying a low-pass filter when generating the envelope) in females in Figures 4 and 7. This agreement suggests that the amplitude criterion is being correctly and consistently applied by visual annotators, but that it fails to consistently isolate power in the slow wave band. Overall, these results provide a rationale for the contrast between the increase in visually annotated N3 sleep and the decrease in frequency-annotated SWS (using SWP) in females.

However, using a frequency-based approach and SWP for labeling SWS still results in males exhibiting less SWS than females (the second of the aforementioned established differences in age-related trends in visually annotated N3 sleep between males and females [37, 38]), and in males exhibiting a steeper decrease in SWS with age than females, as shown in Figure 5. Both of these effects can be explained using the periodograms in Figure 8. With regards to males exhibiting less SWS than females, EEG band power (and, by association, EEG amplitude) is significantly greater in females than in males, meaning females are more likely to meet an absolute SWP (or EEG amplitude) threshold than males. With regards to males exhibiting a steeper decrease in SWS with age than females, males exhibit a broad decrease in EEG band power (and, by association, amplitude) between the ages of 40 and 80 which is not present in females, making males increasingly less likely to meet an absolute SWP (or EEG amplitude) threshold as they age. While an age-related decrease in EEG amplitude is partially reflective of a reduction in synchronous neuronal firing (a key component of slow waves), such a reduction would primarily impact spectral power in the slow wave band, rather than the entire spectrum. A significant contribution to the broad-spectrum reduction in power observed in men between the ages of 40 and 80 is more likely to be from confounding factors such as “differences in homeostatic sleep pressure, brain integrity, and skull thickness” which can reduce the general amplitude of EEG measured at the scalp with age [19, 40].

It is important to recall here that the R & K guidelines were established primarily on young males, meaning sex- and age-related trends in EEG amplitude are unlikely to have been considered when establishing scoring criteria. Indeed, it is well established that a decrease in EEG amplitude with age can lead to N3 epochs being incorrectly scored as N2 epochs [19]. Similarly, that females have a greater EEG amplitude than males has been previously documented [41], with it being “concluded that sex differences in EEG power spectra are not likely to be caused by sex differences in sleep regulatory mechanisms but may, for instance, be caused by sex differences in skull characteristics” [42]. All of these results reinforce that observed sex-based differences in visually annotated N3 sleep may be artifactual rather than physiological, and a result of the 75 µV amplitude criterion.

Compared to the absolute periodograms in Figure 8, the %SWP periodograms in Figure 9 appear to be less affected by sex-based differences in baseline EEG amplitude and how this amplitude evolves with age. In Figure 9, NREM %SWP is comparable between males and females (though slightly greater in females than in males), and age-related changes in the periodograms are consistent between sexes. Indeed, using %SWP for frequency-based annotation of SWS results in a consistent, mild decrease in SWS with age for both sexes and similar amounts of SWS for both males and females (Figures 6 and 7).

Whether these trends (a consistent, mild decrease in SWS with age for both sexes and similar amounts of SWS for both males and females) are correct cannot be directly verified in this study. However, this discussion has reinforced the view that existing sex-based differences in age-related trends in visually annotated N3 sleep are, at least in part, artifacts of the 75 µV criterion. Further, in another study critiquing the amplitude threshold it was noted that “Irrespective of the applied detection (amplitude) threshold..., in the older adults in our data, the number, density..., amplitude, and frequency of slow oscillations were all reduced” [19], implying that an age-related decrease in SWS is expected. Additional evidence exists in animal studies. While most of these studies investigate only male rats [43], looked at age- and sex-related trends in sleep patterns in male and female Fisher-344 rats and concluded that “the effects of aging were comparable in both sexes.” While there are obvious differences between rodent and human sleep patterns and sleep annotation, it is worth noting that rodent sleep annotation employs frequency, rather than amplitude, based criteria.

### Limitations

It should be noted that this analysis is conducted on a single database drawn from a single country and that the age range of the cohort in this database (40–80 years) excludes the “young” 20–30-year-old demographic that is frequently employed in sleep studies [23, 27]. However, the SHHS database is an extremely large and well-established sleep research database, and its size allows for greater granularity in demographic subgroups than might otherwise be possible.

Another limitation of this study is that the SHHS database contains EEG data from only the central C4/A1 and C3/A2 leads (i.e. the ‘acceptable’ AASM EEG derivation for measuring slow waves [15]) as opposed to the ideal frontal F4/A1 or F3/A2 leads. While slow waves are likely to exhibit greater amplitude in the frontal than central leads due to their association with the frontal regions of the brain [44], it has been observed that the “age-related decline in spectral EEG power and amplitude is especially pronounced over frontal areas” [19]. It is therefore likely that similar trends would be observed using frontal (as opposed to central) leads, a premise which is supported both by the AASM acceptance of using the C4/A1 and C3/A2 leads to measure slow waves, and by the results in this manuscript being consistent with those previously reported in similar studies on smaller cohorts in the literature [23, 41, 42].

A further limitation of this work is that the thresholds used for frequency-based labeling of SWS are calibrated using visually annotated N2 and N3 epochs, meaning that these thresholds are dependent on existing visual annotation criteria (which include an amplitude criterion). As mentioned previously, this approach was selected as the only reference labels available in the SHHS dataset are visual annotations. To characterize the sensitivity of age- and sex-related trends in frequency-labeled SWS to the selected thresholds, a sensitivity analysis (Appendix D) was conducted. This analysis showed that the majority of observed age- and sex-related trends remained consistent when the SWS labeling threshold was reduced (to 90% of optimal) or increased (to 110% of optimal). Furthermore, it is important to emphasize that these frequency-based labeling methods were developed to highlight how different elements of the AASM guidelines for labeling slow waves affect age- and sex-related trends in SWS, and to suggest future avenues for reviewing these guidelines, but not necessarily to serve as a future method for labeling slow waves. It is worth noting here that methods do exist in the literature for the automated labeling of slow waves from EEG ( [23, 34],). These methods typically detect a variety of fiducial points from the EEG, using the AASM (along with supplemental) thresholds to label slow waves, and often facilitate and indeed recommend that amplitude thresholds be reduced for older participants. However, none of these methods have yet seen widespread adoption or facilitated a review of the AASM guidelines for labeling slow waves.

## Conclusion

The results in this manuscript highlight that the amplitude threshold in current AASM rules for labeling N3 sleep may produce artifactual age-related trends in females as compared to males (as primarily data from males were used to establish the scoring criteria).The scoring guidelines for N3 sleep should be revisited, and their efficacy and appropriateness evaluated over a large, diverse cohort. While it is important to acknowledge the existing use of these sleep scoring guidelines and to maintain comparability with prior work, these advantages do not outweigh the disadvantages of the current guidelines, which potentially introduce biased results in female subjects, and possibly elderly patients. New guidelines, ideally incorporating frequency-based information, should be derived to ensure equal sleep staging performance regardless of sex or age.

## Supplementary material

Supplementary material is available at *SLEEP* online.

zsaf063_suppl_Supplementary_Materials

## Data Availability

The data underlying this article are available in the National Sleep Research Resource (NSRR), at https://dx.doi.org/10.1093/jamia/ocy064. The datasets were derived from sources in the public domain: https://sleepdata.org/.
