# Supplementary material for: Is it time to revisit the scoring of slow wave (N3) sleep?
Source: Sleep. 2025 Mar 13;48(10):zsaf063. doi: 10.1093/sleep/zsaf063 (PMC12515605; doi:10.1093/sleep/zsaf063)
Supplement: zsaf063_suppl_Supplementary_Materials [file zsaf063_suppl_supplementary_materials.pdf]

Supplemental Material for: Is it time to revisit the scoring of Slow Wave (N3) Sleep?

Shaun Davidson[1*], Rachel Sharman[2], Simon D Kyle[2], Lionel Tarassenko[1]

[1]Institute of Biomedical Engineering, Department of Engineering Science, University of Oxford

[2]Sir Jules Thorn Sleep and Circadian Neuroscience Institute, Nuffield Department of Clinical Neurosciences, University of Oxford

Appendix A. Removal of Noisy C4/A1 EEG Records

In certain participants in the SHHS1 database with noisy C4/A1 EEG recordings, sleep annotation was performed solely using the C3/A2 EEG. As such, it was important to identify and remove these recordings from the selected cohort of SHHS1 participants. These records were identified by assessing the following metrics:

- The per-participant ratio between mean (C4/A1) SWP during visually-annotated N3 sleep and mean (C4/A1) SWP during visually-annotated N2 sleep, which is expected to be greater than 1.
- The per-participant ratio between mean (C4/A1) SWP during visually-annotated N3 sleep and mean (C3/A2) SWP during visually-annotated N3 sleep, which should not be significantly less than 1.

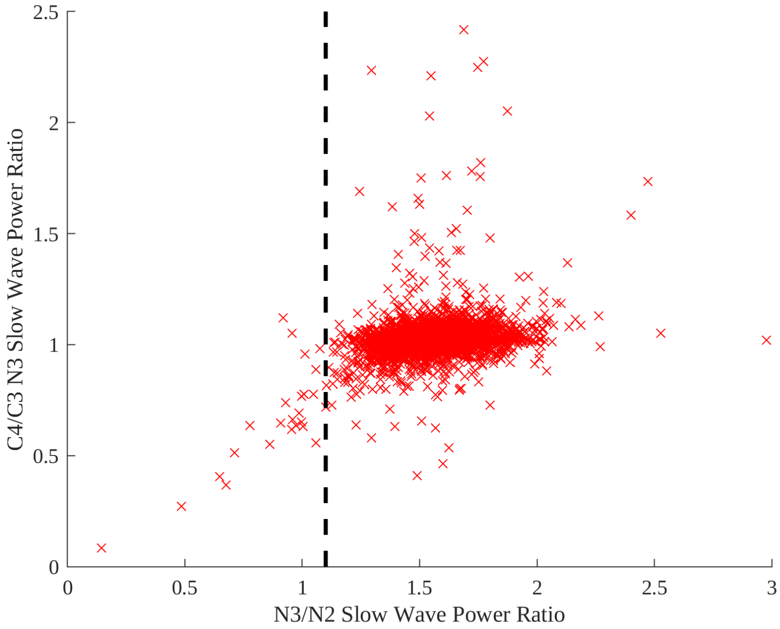

Figure A1. Scatter plot of ratio between (C4/A1) N3 SWP and (C4/A1) N2 SWP (x-axis) and ratio between (C4/A1) N3 SWP and (C3/A2) N3 SWP (y-axis), with SQI cut off represented by dashed line.

Fig. A1 shows a scatter plot these two metrics for the selected cohort of SHHS1 participants. This figure shows there is a group of records where the N3/N2 SWP ratio

is less than 1 for the C4/A1 EEG and N3 SWP is significantly higher for the C3/A2 EEG than C4/A1 EEG (i.e., records in the lower-left corner of the plot). These records are likely those with low quality C4/A1 EEG where sleep annotation was performed solely using the C3-A2 EEG. As such, recordings with a C4/A1 EEG N3/N2 slow wave power ratio of less than 1.1 (the dotted line in fig. A1) were removed from the cohort.

Appendix B. Additional Cohort Demographic Information

Tables B1 and B2 show age- and sex-related trends in the Apnoea-Hypopnoea Index (AHI) and Arousal Index (AI), respectively, in the selected cohort of SHHS1 participants. AHI increases with age in both males and females until the 70 - 80 age band, which exhibits a very similar AHI to the 60 - 70 age band. Further, males exhibit a consistently greater mean AHI than females, as reported in the literature [36].

Table B1. AHI grouped by sex and age for the selected cohort of SHHS1 participants. All values presented as mean (standard deviation).

Age	Males	Females	Overall
40 - 50 years	8.01 (3.81)	5.57 (3.74)	6.48 (3.94)
50 - 60 years	8.51 (3.84)	6.39 (3.80)	7.11 (3.94)
60 - 70 years	8.78 (3.75)	7.70 (3.95)	8.08 (3.91)
70 - 80 years	8.73 (3.75)	7.87 (3.62)	8.18 (3.69)

In table B2, AI increases consistently with age in both males and females until the 70 - 80 age band, which exhibits a very similar AI to the 60 - 70 age band. Further, males exhibit a slightly greater mean AI than females in each age band.

Table B2. AI grouped by sex and age for the selected cohort of SHHS1 participants. All values presented as mean (standard deviation).

Age	Males	Females	Overall
40 - 50 years	14.3 (6.5)	13.3 (5.8)	13.7 (6.1)
50 - 60 years	15.7 (6.6)	14.1 (5.9)	14.6 (6.2)
60 - 70 years	16.1 (7.4)	14.9 (6.4)	15.4 (6.8)
70 - 80 years	15.8 (6.9)	15.1 (7.4)	15.3 (7.2)

Of the 2,913 SHHS1 participants in the selected cohort, 2,511 (86.2%) responded to an interim follow up questionnaire 1 - 2 years after the SHHS1 study. Of these 2,511 participants, 41 stated they had been diagnosed with a sleep disorder other than sleep apnoea, while 14 responded that they “didn’t know”. A further follow

up questionnaire was conducted during the SHHS2 study (approximately 5 years after SHHS1), to which 2,207 (75.8%) of the selected cohort of SHHS1 participants responded. Of these 2,207 participants, 57 reported having been diagnosed with a sleep disorder other than sleep apnoea, with 26 reporting a diagnosis of insomnia, 25 a diagnosis of restless leg syndrome, 18 “other”, and 0 narcolepsy (note that participants could report being diagnosed with multiple conditions).

Appendix C. Selecting Thresholds for Labelling SWS

Fig. C1 shows the overall percentage of N2 and N3 epochs meeting AASM definitions when different envelope amplitude thresholds are used to label SWS across the 2,913 patient cohort of SHHS1 recordings. Performance peaks with 87.0% of epochs meeting AASM definitions at a SWP threshold of 18 µV.

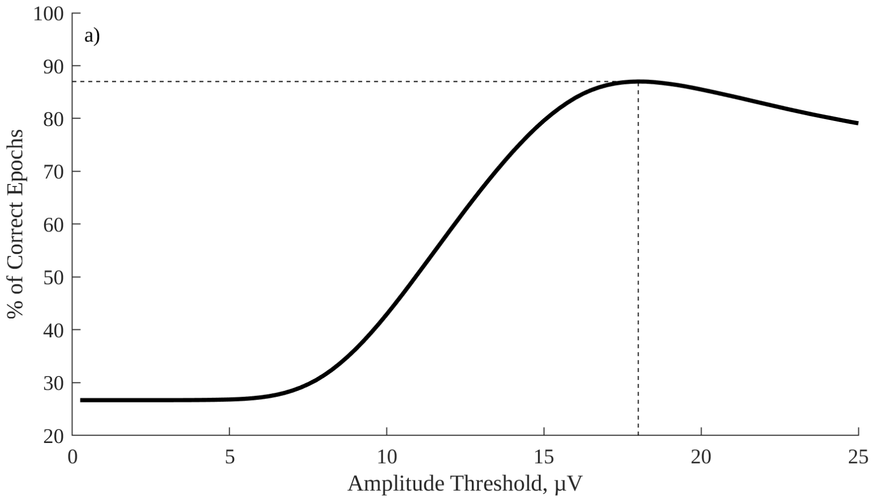

Figure C1. % of epochs meeting AASM definitions when different envelope amplitude thresholds are used for labelling SWS across the selected cohort of SHHS1 patients.

Fig. C2 shows the overall percentage of N2 and N3 epochs meeting AASM definitions when different SWP thresholds are used to label SWS across the selected cohort of SHHS1 recordings. Performance peaks with 84.8% of epochs meeting AASM definitions at a SWP threshold of 285 μV^2.

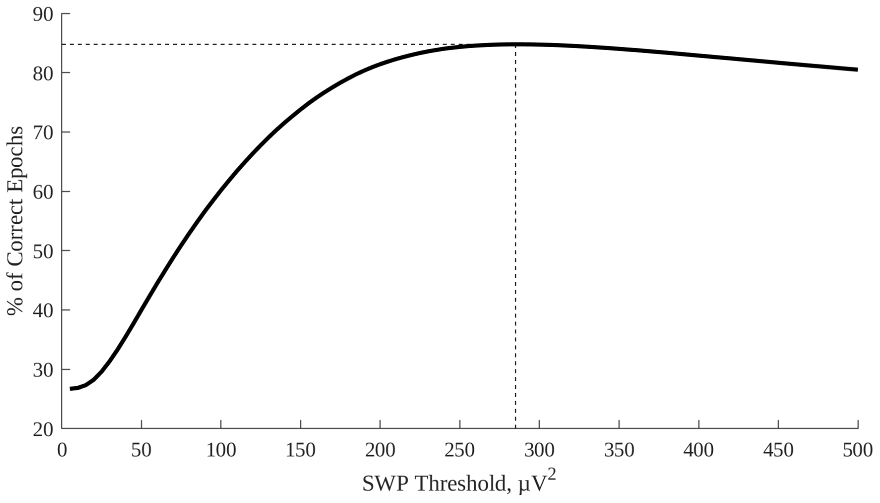

Figure C2. % of epochs meeting AASM definitions when different SWP thresholds are used for labelling SWS across the selected cohort of SHHS1 patients.

Fig. C3 shows the overall agreement between visual and frequency-based annotation of N2 and N3 epochs peaks at 81.4% and a threshold of 70% SWP. Peak agreement with visual annotation being lower when using %SWP than envelope amplitude (87.0%, fig. C1) or absolute SWP (84.8%, fig. C2) is expected given the stronger association between the previous methods and EEG amplitude (as employed in visual annotation).

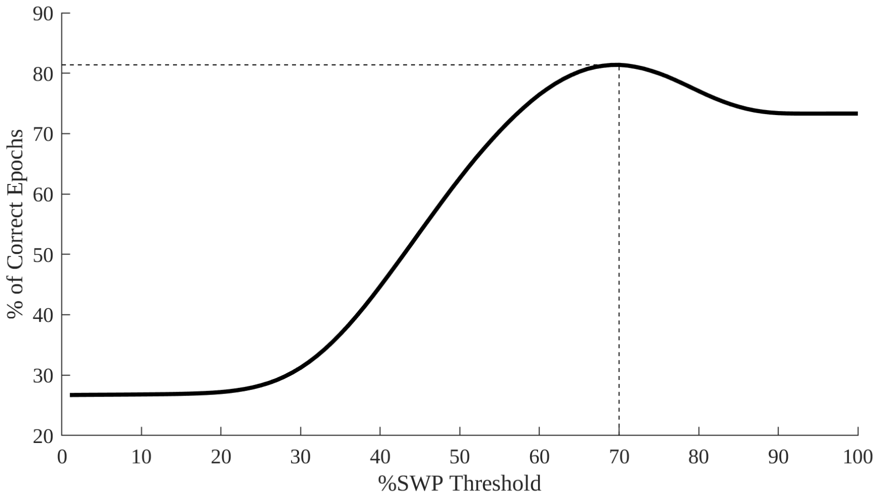

Figure C3. % of epochs meeting AASM definitions when different %SWP thresholds are used for labelling SWS across the selected cohort of SHHS1 patients.

Appendix D. Threshold Sensitivity Analysis

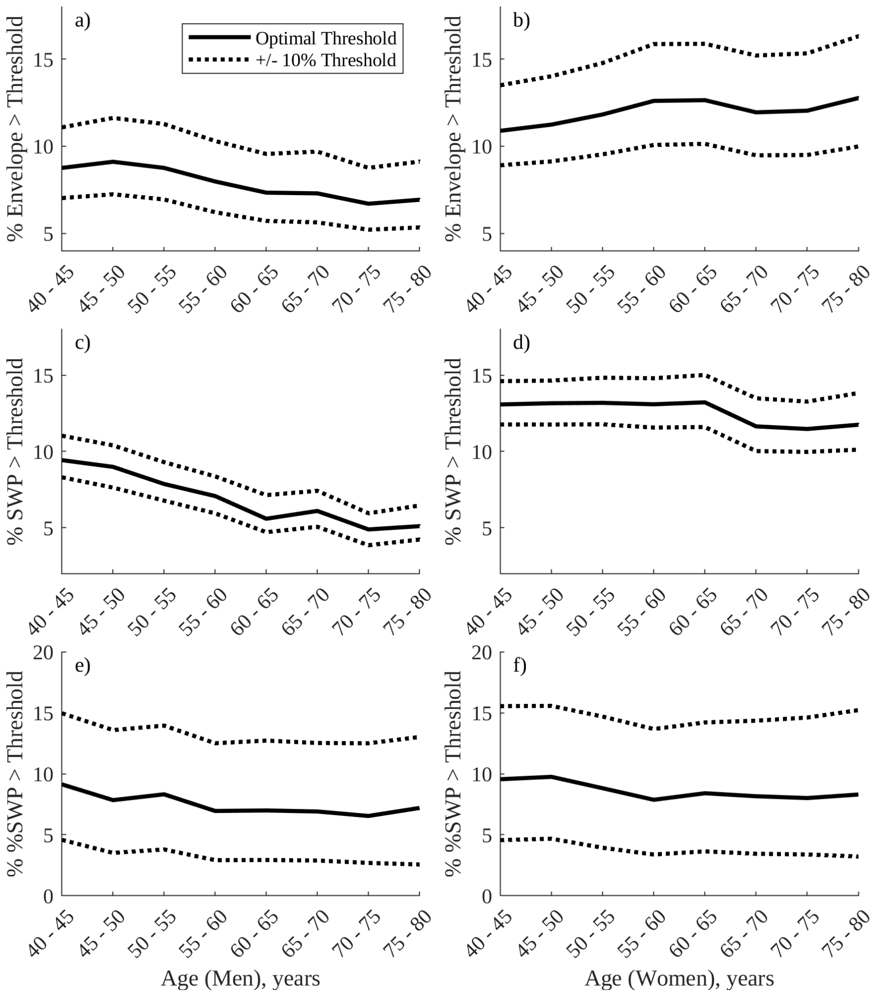

Figure D1. Age-related trends in amplitude- and frequency-labelled SWS using different thresholds for labelling, grouped by age for males and females in the SHHS1 database: a) % overnight duration of envelope amplitude > threshold, males; b) % overnight duration of envelope amplitude > threshold, females; c) % overnight duration of SWP > threshold, males; d) % overnight duration of SWP > threshold, females; e) % overnight duration of %SWP > threshold, males; f) % overnight duration of %SWP > threshold, females.

Fig. D1 and table D1 show how age-related trends in amplitude- and frequency-labelled SWS are affected by using a threshold of 10% less than or 10% greater than the optimal threshold selected in the paper. As would be expected, using a lower threshold results in an increase in amplitude- or frequency-labelled SWS and using a higher threshold a decrease. SWS labelled using SWP (panels c and d) varies the least as a result of this

10% change in labelling threshold, while SWS labelled using %SWP (panels e and f) varies the most. This behaviour is likely due to %SWP being normalised to a range of 0 - 100 (as compared to envelope amplitude and SWP, which are not normalised), and thus having a narrower underlying distribution. Indeed, similar behaviour can be observed in the plots in Appendix C, where the % of epochs meeting AASM definitions varies little when using a SWP threshold (fig. C2), but moderately when using an envelope amplitude (fig. C1) or %SWP (fig. C3) threshold over the range of threshold values employed in this sensitivity analysis.

Table D1. Linear trend statistics for automatically-labelled SWS using different thresholds in fig. D1

**Metric**	$\mathbf{R^2}$	**p**	**Constant**	**Gradient**
%Envelope amplitude > 16.2 µV, males	0.85	1.10E-3	11.97	-0.40
%Envelope amplitude > 16.2 µV, females	0.69	0.01	13.63	0.33
%Envelope amplitude > 18 µV, males	0.89	4.73E-4	9.46	-0.36
%Envelope amplitude > 18 µV, females	0.57	0.03	11.05	0.21
%Envelope amplitude > 19.8 µV, males	0.90	2.95E-4	7.58	-0.31
%Envelope amplitude > 19.8 µV, females	0.37	0.11	9.10	0.11
%SWP > 256.5 μV^2, males	0.93	1.06E-4	11.53	-0.73
%SWP > 256.5 μV^2, females	0.49	0.05	15.17	-0.19
%SWP > 285 μV^2, males	0.92	1.42E-4	9.97	-0.69
%SWP > 285 μV^2, females	0.67	0.01	13.77	-0.26
%SWP > 313.5 μV^2, males	0.93	1.26E-4	8.70	-0.64
%SWP > 313.5 μV^2, females	0.76	4.88E-3	12.45	-0.31
%SWP > 63%, males	0.58	0.03	14.47	-0.28
%SWP > 63%, females	0.11	0.43	15.15	-0.09
%SWP > 70%, males	0.65	0.02	8.79	-0.29
%SWP > 70%, females	0.61	0.02	9.62	-0.23
%SWP > 77%, males	0.80	2.75E-3	4.36	-0.25
%SWP > 77%, females	0.79	3.23E-3	4.69	-0.20

Overall trend topography and direction are preserved for all labelling methods in fig. D1 and table D1 across the range of thresholds employed. SWS as labelled using envelope amplitude shows a consistent increase with age in females for all thresholds,

and all other metrics show a consistent decrease with age for all thresholds. In table D1, two metrics do not exhibit statistically significant linear trends: % envelope amplitude > 19.8 μV in females (the higher threshold) and % SWP $> 63\%$ in females (the lower threshold).
